# Supplementary material for: COVID-19 Testing Strategies for K-12 Schools in California: A Cost-Effectiveness Analysis
Source: Int J Environ Res Public Health. 2022 Jul 30;19(15):9371. doi: 10.3390/ijerph19159371 (PMC9367893; doi:10.3390/ijerph19159371)
Supplement: Supplementary file 1 [file ijerph-19-09371-s001.zip › ijerph-1806604-supplementary.pdf]

Supplement S1. Model visualization and medical costs.

Model design

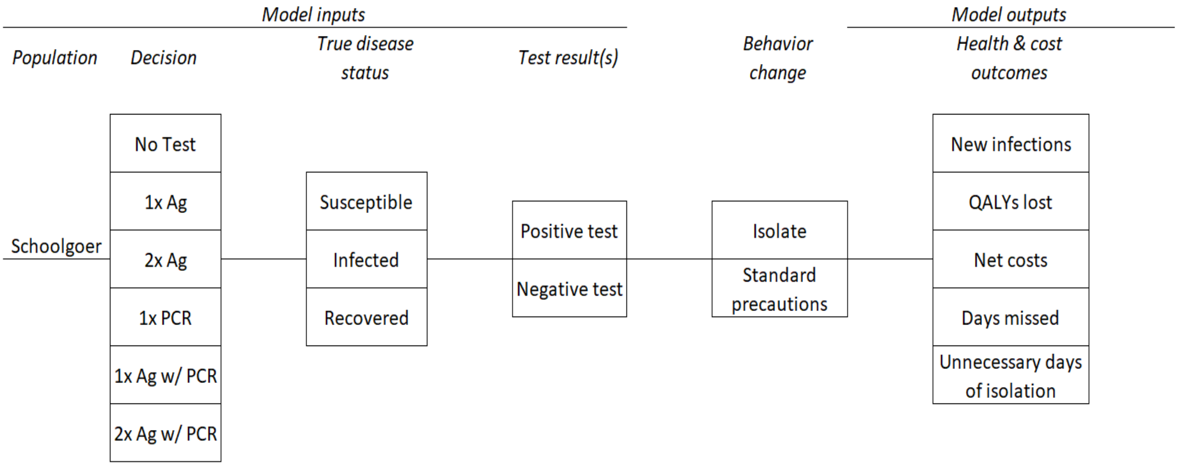

Figure S1. Simplified decision tree model. Each box branches out to the following set of boxes.

Cost of COVID-19 treatment

The cost of treating acute COVID-19 depends on severity. While asymptomatic individuals will not require any treatment and generate no costs, severely ill individuals require critical care and incur very high costs. We used the distribution of disease severity among those infected with COVID-19 and the respective average costs incurred for each level of severity to calculate a weighted average cost of treatment (Table S1).

Table S1. Breakdown of treatment costs by type of care sought. ED: emergency department; ICU: intensive care unit.

Type of care needed	% of infected	Cost
**Does not seek care**	79%	USD 0
**Seeks outpatient care**	2%	USD 513
**Seeks care at ED**	4%	USD 815
**Requires hospitalization**	12%	USD 12,000
**Requires ICU admission**	3%	USD 61,000
**Weighted total**	**100%**	**USD 3,312**

Supplement S2. Dynamic approaches to testing.

Findings from our main study suggest that screening strategies should be responsive to changes in the epidemiology of COVID-19. We conducted several sensitivity analyses to explore what circumstances might allow relaxation or abandonment of screening protocols and what circumstances might require more intensive screening.

Our model does not incorporate temporal effects of transmission rate on community prevalence (i.e., even when R_{eff} increases, e.g., due to a higher transmission variant, prevalence does not automatically change as a result, or vice versa). To overcome this, we conducted two-way sensitivity analyses to visualize the relationship between screening outcomes and both

prevalence and transmission rate simultaneously. Of note, we assumed that the community-level protection against SARS-CoV-2 (i.e., the proportion of individuals with some degree of immunity) that existed at the beginning of the Omicron surge would be retained over time, and we kept this value at 86% for the analyses described below. While individual-level immunity may wane, we presume that additional booster shots and recurring infections will keep the population average relatively stable and prevent substantial decreases in herd immunity. Despite this stable level of immunity, the transmission rate can change according to viral characteristics.

Figure S2 is a two-way sensitivity analysis. It depicts the change in net cost when twice weekly antigen testing with PCR follow up is conducted as opposed to once weekly. The lines represent different R_{eff} and the horizontal axis represents the prevalence of the virus. As noted above, a higher R_{eff} may lead to a higher prevalence. Positive values mean that once weekly testing is less expensive, negative values reflect net savings of twice weekly over once weekly. Since more frequent testing always prevents more infections than less frequent testing, 2xAg/PCR is dominant over 1xAg/PCR (fewer costs *and* fewer transmissions) whenever the change in net cost in Figure S2 is below zero. For example, a community with $R_{eff} = 1.5$ would save costs and improve health by ramping up testing to twice weekly once the prevalence of infection reaches 4%. If the transmission rate is greater, a lower prevalence would be enough to warrant increasing testing frequency.

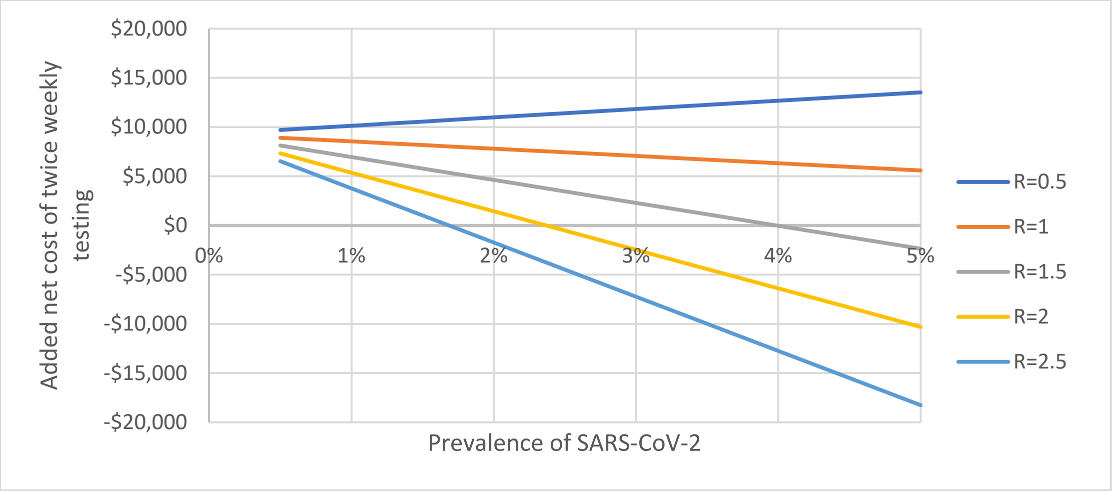

Figure S2. Added net costs of 2xAg/PCR compared to 1xAg/PCR.

On the other hand, when transmission rate is low (R_{eff}<1) implementing 2xAg/PCR increases net costs over 1xAg/PCR. When few transmissions are occurring, twice weekly testing does not capture enough infections (and thereby does not prevent enough medical costs) to offset the added cost of testing or the additional productivity loss. While not dominant, twice weekly remains cost-effective (based on a conventional threshold of <USD 100,000 per QALY gained) when the transmission rate is high (Figure S3).

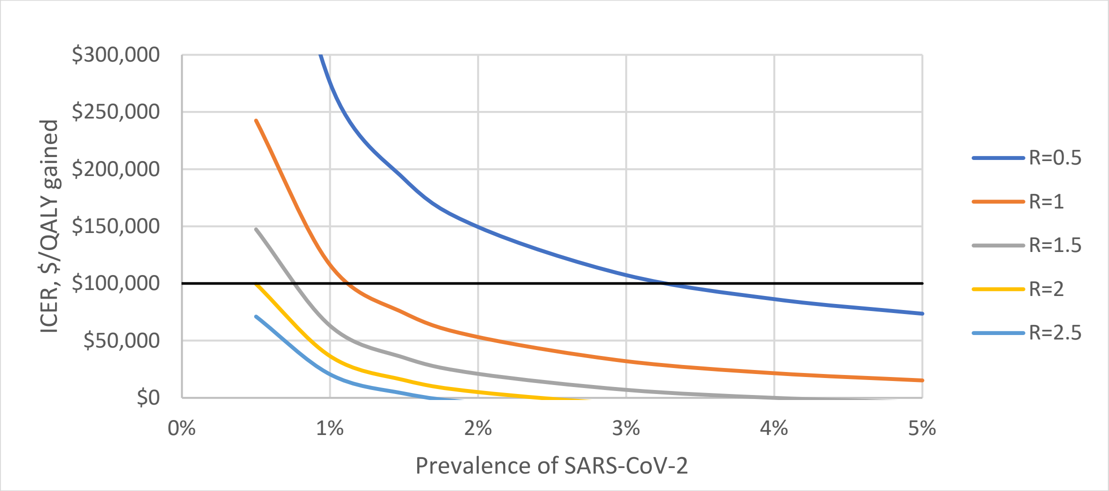

Figure S3. Incremental cost-effectiveness ratio of 2xAg/PCR versus 1xAg/PCR. Y-axis capped at USD 300,000/QALY gained.

Figure S4 compared 1xAg/PCR to no test. 1xAg/PCR leads to greater cost savings over no test with increasing transmission rate (Figure S4), and always prevents more transmissions than no testing. It is therefore dominant whenever savings occur (e.g., in high prevalence settings with low transmission rate or high transmission rate in low prevalence settings). When 1xAg/PCR incurs greater net costs than no test, it is cost-effective in most circumstances (<USD 100,000 per QALY gained), mainly when prevalence is greater than 1% with R_{eff}=1, or when prevalence is lower with greater R_{eff} values (Figure S5). When both transmission rate and prevalence is low, even though screening will continue to prevent transmissions, the benefit may be so low that ICERs may become prohibitive, such as with R_{eff}=0.5 and prevalence of 1% which leads to an ICER of USD 170,000 per QALY gained.

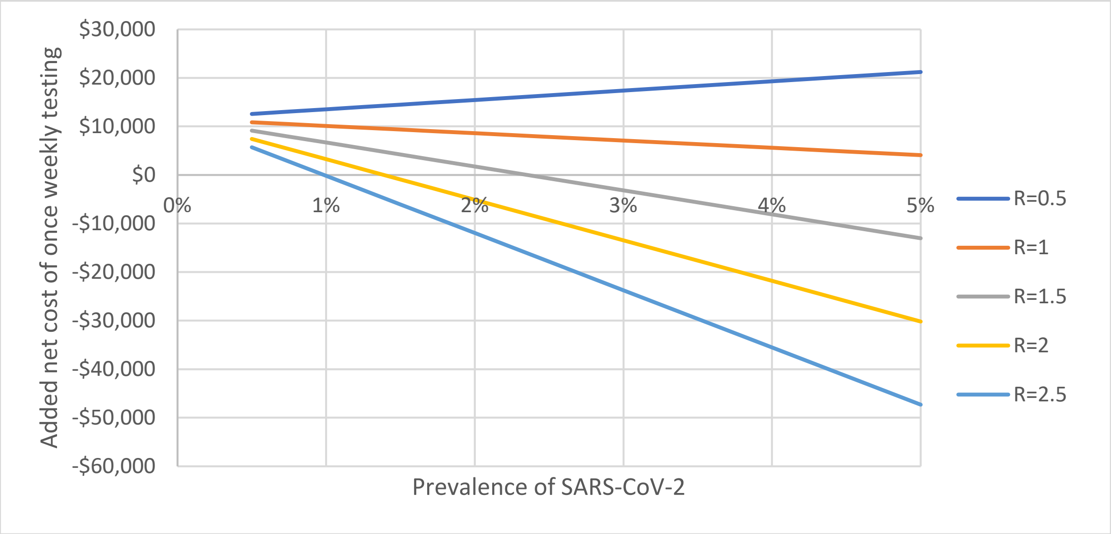

Figure S4. Added net costs of 1xAg/PCR compared to no testing.

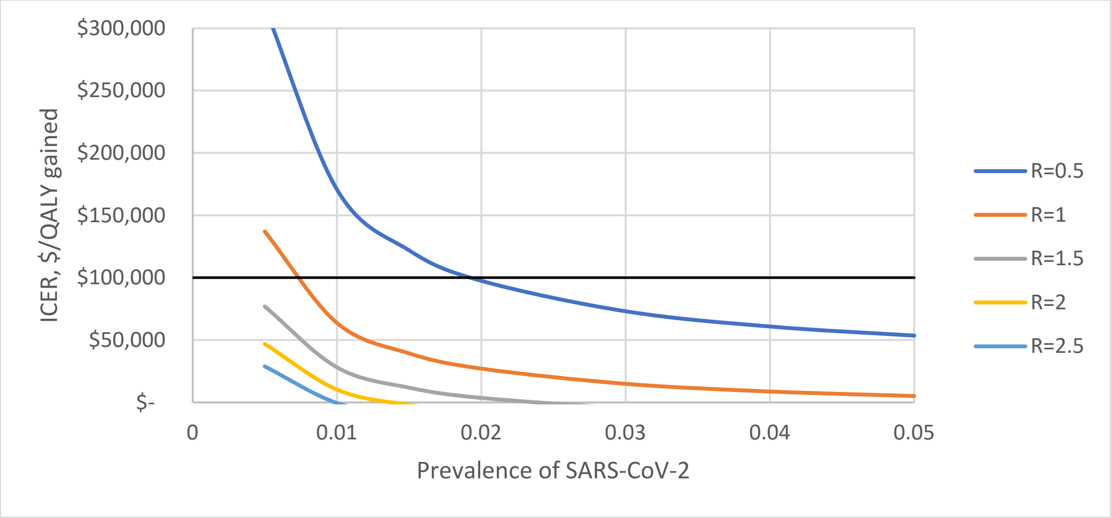

Figure S5. Incremental cost-effectiveness ratio of 1xAg/PCR versus no testing. Y-axis capped at USD 300,000/QALY gained.

In circumstances where both 1xAg/PCR versus no test and 2xAg/PCR versus 1xAg/PCR are cost-effective (ICER for the latter being larger than the ICER for the former), it may be reasonable to focus resources on disproportionately vulnerable communities so that more cost-intensive strategies can be implemented to achieve equitable outcomes. For example, historically underserved, low-income populations may be more likely to both contract COVID-19 and to experience poorer outcomes if they do, as well as incur greater financial costs due to illness. Schools that serve such populations might benefit from implementing more frequent screening testing, and policymakers may consider making these locations a priority when distributing limited resources. As COVID-19 transitions to endemic status, decisionmakers must identify what levels of risk are acceptable in their communities to decide when to stop school-based screening entirely.
